# Bifunctional Paramagnetic and Luminescent Clays Obtained by Incorporation of Gd^3+^ and Eu^3+^ Ions in the Saponite Framework

**DOI:** 10.1021/acs.inorgchem.1c01455

**Published:** 2021-07-09

**Authors:** Stefano Marchesi, Chiara Bisio, Daniela Lalli, Leonardo Marchese, Carlos Platas-Iglesias, Fabio Carniato

**Affiliations:** † Dipartimento di Scienze e Innovazione Tecnologica, Università degli Studi del Piemonte Orientale “Amedeo Avogadro”, Viale Teresa Michel 11, 15121 Alessandria, Italy; ‡ CNR-SCITEC Istituto di Scienze e Tecnologie Chimiche “G. Natta”, Via C. Golgi 19, 20133 Milano, Italy; § Centro de Investigacións Científicas Avanzadas (CICA) and Departamento de Química, Facultade de Ciencias, Universidade da Coruña, 15071 A Coruña, Galicia, Spain

## Abstract

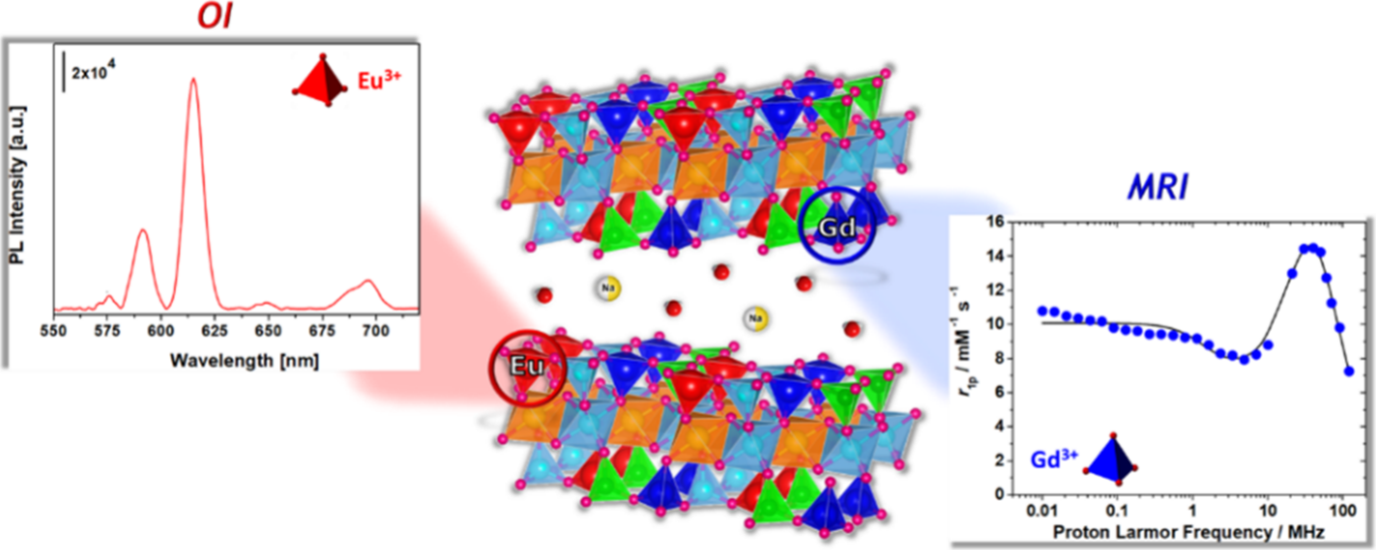

A novel bifunctional saponite clay incorporating gadolinium (Gd^3+^) and europium (Eu^3+^) in the inorganic framework was prepared by one-pot hydrothermal synthesis. The material exhibited interesting luminescent and paramagnetic features derived from the co-presence of the lanthanide ions in equivalent structural positions. Relaxometry and photoluminescence spectroscopy shed light on the chemical environment surrounding the metal sites, the emission properties of Eu^3+^, and the dynamics of interactions between Gd^3+^ and the inner-sphere water placed in the saponite gallery. The optical and paramagnetic properties of this solid make it an attractive nanoplatform for bimodal diagnostic applications.

## Abstract 2

The combination of gadolinium and europium with synthetic saponite clays leads to the preparation of novel lanthanide-based multifunctional layered platforms, with interesting paramagnetic and optical features exploitable for the development of new systems for luminescent devices and in magnetic/optical diagnostics.

## 1 Introduction

In the last decade, there has been large interest in the optimization and employment of synthetic clays for scientific and technological applications.^1−15^ In particular, synthetic saponite clays have been studied for their interesting properties, in terms of high thermal stability, specific surface area, tunable acidity, low costs, and excellent chemical versatility.^1−3^

Their chemical properties can be easily tuned by modifying the particle size^2^ and/or chemical composition of the interlayer space^4^ and the inorganic framework.^5,6^ These modifications can be achieved using specific post-synthetic treatments (*i.e.*, intercalation of cationic organic and inorganic compounds) or properly modifying the synthesis method and selecting appropriate precursors for one-pot syntheses.

On this basis, with a proper choice of the intercalated entities (*i.e.*, metal ions, dyes, ...) or by modifying the synthetic protocols, it is possible to design new functionalized saponites with innovative features suitable for different applications.^13−20^ In particular, the introduction of one or more f-block elements (*i.e.*, Gd^3+^, Eu^3+^, Tb^3+^, ...), in the form of ions or complexes, allows preparing novel versatile materials owning the chemical and mechanical robustness of saponite clays and the peculiar luminescent and magnetic properties conferred by the metal ions. While the luminescent features make such clays suitable for a variety of industrial applications as chemical sensors, luminescent thermometers, or in bioimaging fields, their magnetic properties are exploited in the medical fields, since they render the materials suitable as probes for different diagnostic imaging modalities.

Recently, we reported the intercalation of positively charged paramagnetic Gd^3+^-chelates, with different hydration states, within the interlayer space of saponite clays, following a common post-synthetic procedure.^4^ The in-depth analysis of the relaxometric properties of such functionalized solids allowed access to the chemical environment of the confined chelates and to the water diffusion processes within the interlayer space.^4^

Lanthanide ions can also be directly included in the inorganic framework of saponite during the gel synthesis. This alternative strategy was tested in our lab embedding in the tetrahedral layer two luminescent Tb^3+^ and Eu^3+^ ions through a modified *one-pot* hydrothermal procedure. The material exhibited noteworthy photophysical features, due to the occurrence of a Tb^3+^–Eu^3+^ energy transfer mechanism, and interesting detection properties for chromate anions in water.^21^

The one-pot preparation of the inorganic framework shows important advantages with respect to the post-synthesis intercalation, such as low production costs, shorter synthesis times, and high chemical stability, due to the lack of lanthanide leaching.

**Figure 1:**
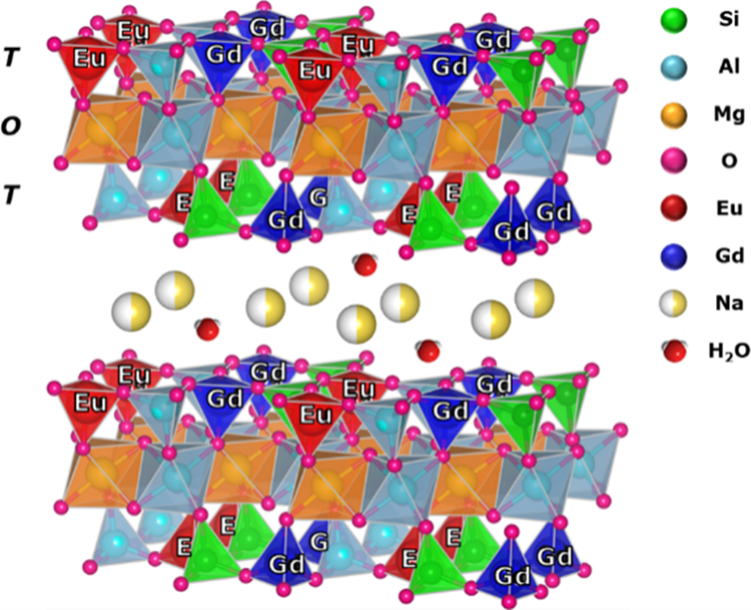
Representative schematic structure of the Na-GdEuSAP sample.

Here, Eu^3+^ and Gd^3+^ ions were individually and simultaneously incorporated for the first time in the framework of a nanosized synthetic clay by means of the above-quoted *one-pot* hydrothermal procedure.^22−24^ The new material containing Gd^3+^ and Eu^3+^ sites in the framework position is hereafter named as Na-GdEuSAP (Figure 1); the materials containing only Gd^3+^ or Eu^3+^ sites in the framework position and without the lanthanide ions, prepared as references, are named as Na-GdSAP, Na-EuSAP, and Na-SAP, respectively.

From one side, Eu^3+^ was selected as a luminescent probe due to its well-defined emission profile in the visible range, high photostability, and long luminescence lifetimes (from micro to ms).^25^ Moreover, Gd^3+^ ions characterized by a great magnetic moment (7.9 Bohr magneton) and suitable electronic relaxation times (10^–9^ to 10^–11^ s) were selected as magnetic resonance imaging reporters because they are able to enhance the longitudinal relaxation rate (*R*_1_) of surrounding H_2_O protons.^26^

A detailed multitechnique physicochemical characterization was performed for all the prepared materials, devoting particular attention to the investigation of their photophysical and relaxometric properties.

## 2 Experimental Section

### 2.1 Materials

#### 2.1.1 Synthesis of Na-GdEuSAP

The synthetic saponite clay, containing both Gd^3+^ and Eu^3+^ ions, was prepared with a modified *one-pot* hydrothermal method adapted from the literature (Scheme S1).^22−24^ A gel with the molar composition of [SiO_2_/MgO/Al_2_O_3_/Na_2_O/GdCl_3_/EuCl_3_/H_2_O] 1:0.835:0.056:0.056:0.01:0.01:20 and H_2_O/Si molar ratio of 20 was prepared. In detail, 6.68 g (0.10 mol) of amorphous silica (SiO_2_ fumed, 99.8%) was gradually dispersed in a solution prepared by dissolving 0.63 g (0.01 mol) of sodium hydroxide (NaOH) in 45.00 g (2.50 mol) of ultrapure water (equal to 5/6 of the total water content). The obtained gel was then mixed accurately. After 30 min, 24.86 g (0.09 mol) of magnesium acetate tetrahydrate [Mg(CH_3_COO)_2_·4H_2_O, 99%] and 3.20 g (0.01 mol) of aluminum isopropoxide {Al[OCH-(CH_3_)_2_]_3_, 98%} were added to the reaction mixture. In parallel, in a second flask, a suspension composed of anhydrous gadolinium chloride (GdCl_3_; 0.373 g, 0.001 mol), europium chloride (EuCl_3_; 0.353 g, 0.001 mol), tetraethyl orthosilicate (6 mL, 0.03 mol), and 0.50 g of ultrapure water was stirred for 10 min at room temperature (RT), and the pH was corrected between 2 and 3 with 1–2 drops of concentrated sulfuric acid (H_2_SO_4_). This suspension was then mixed with the gel containing aluminum isopropoxide, magnesium acetate, and silica. The remaining ultrapure water (5.00 g, 0.28 mol) was then added to the reaction mixture. After 4 h, the gel, with a pH between 8 and 9, was introduced in a Teflon cup (125 mL capacity) of an autoclave (Anton Paar 4748) and heated in an oven for 72 h at 240 °C. After hydrothermal treatment, the product was filtered, washed with hot ultrapure water up to neutral pH, and dried in an oven overnight at 100 °C. The as-produced material (called as GdEuSAP) was submitted to a cation-exchange procedure in order to ensure the chemical uniformity of the exchange sites. In detail, 2.50 g of GdEuSAP was dispersed in 250 mL of saturated sodium chloride (NaCl) solution for 36 h at RT to replace the cations present in the interlayer space (*i.e.*, Al^3+^, Mg^2+^, and H^+^) with Na^+^. The final solid (Na-GdEuSAP) was filtered, washed with hot ultrapure water until complete elimination of chlorides (confirmed by a silver nitrate spot test), and finally dried in an oven overnight at 100 °C.

#### 2.1.2 Synthesis of Na-GdSAP and Na-EuSAP Clays

The preparation of nanosized Na-GdSAP and Na-EuSAP samples followed the same procedure described above.

#### 2.1.3 Synthesis of Na-SAP Clays

Nanosized synthetic saponite with a cationic exchange capacity (CEC) of 87.9 ± 2.3 mequiv/100 g was synthesized following the classical hydrothermal method.^1^ A gel with the molar composition of [SiO_2_/MgO/Al_2_O_3_/Na_2_O/H_2_O] 1:0.835:0.056:0.056:20 and H_2_O/Si molar ratio of 20 was prepared. In detail, 11.91 g (0.19 mol) of fumed SiO_2_ (99.8%) was gradually dispersed in a solution prepared by dissolving 0.93 g (0.02 mol) of NaOH in 58.20 g (3.23 mol) of ultrapure water. The obtained gel was then mixed accurately. After 1 h, 37.78 g (0.18 mol) of Mg(CH_3_COO)_2_·4H_2_O (99%) and 4.71 g (0.02 mol) of Al[OCH(CH_3_)_2_]_3_ (98%) were added to the reaction mixture, along with the remaining ultrapure water (16.36 g, 0.91 mol). After 2 h, the gel was introduced in a Teflon cup of an autoclave and heated in an oven for 72 h at 240 °C. After hydrothermal treatment, the product was filtered, washed with hot ultrapure water up to neutral pH, and dried in an oven overnight at 100 °C. The as-produced material, called as SAP, was submitted to a cation-exchange procedure in order to ensure the chemical uniformity of the exchange sites. A total of 2.50 g of SAP was dispersed in 250 mL of saturated NaCl solution for 36 h at RT. The final solid (named as Na-SAP) was filtered, washed with hot ultrapure water until complete elimination of chlorides, and finally dried in an oven overnight at 100 °C.

### 2.2 Experimental Methods

The elemental analyses were performed on a Thermo Fisher Scientific X5 Series inductively coupled plasma mass spectrometer (ICP-MS) (Waltham, MA, USA). Prior to the analyses, the solids were mineralized by treatment with a mixture of nitric acid (HNO_3_, 5 mL) and hydrofluoric acid (HF, 5 mL) at 100 °C for 8 h.

X-ray powder (XRPD) diffractograms were collected with a ThermoARL X’TRA-048 powder diffractometer with a Cu K_α1_ (λ = 1.54062 Å) monochromatic radiation. Diffractograms were recorded at RT in the 2–65° 2θ range with a step size of 0.02° and a rate of 1.0°/min. The X-ray profiles at low angles (2–15° 2θ) were collected with narrower slits and a rate of 0.25°/min.

High-resolution transmission electron microscopy (HRTEM) micrographs were collected on a Zeiss libra200 FE3010 high-resolution transmission electron microscope operating at 200 kV. Specimens were prepared by depositing the samples on carbon-coated grids.

The CEC parameter was determined by the ultraviolet–visible (UV–Vis) method reported in the literature.^2^ 0.300 g of Na-SAP was exchanged with 10 mL of 0.02 M solution of [Co(NH_3_)_6_]^3+^ at RT for 60 h. After separation by centrifugation (5000 rpm for 5 min, two times), the solution was analyzed by UV–Vis spectroscopy. UV–Vis spectra were recorded at RT in the range 300–600 nm with a resolution of 1 nm, using a double-beam PerkinElmer Lambda 900 spectrophotometer. The absorbance of the band at 475 nm (^1^A_1g_ → ^1^T_1g_), relative to a d–d spin-allowed Laporte-forbidden transition of Co^3+^, was evaluated to quantify the amount of Co^3+^ ions free in solution.

Dynamic light scattering (DLS) experiments were carried out at 25 °C using a Malvern Zetasizer Nano ZS operating in a particle size range from 0.6 nm to 6 mm and equipped with a He–Ne laser with λ = 633 nm. The samples were dispersed in ultrapure water (5 mg in 3 mL) in the presence of xanthan gum (0.1 wt %) to improve particle dispersion. Before measurements, the suspensions were sonicated for 10 min. The particles dispersed in water tend to form large aggregates. In the solution stabilized with xanthan gum, this effect is strongly limited, and no precipitation is observed after hours. The pH of suspensions was 7.0.

Photoexcitation and photoluminescence (PL) emission spectra were recorded on a HORIBA Jobin Yvon model IBH FL-322 Fluorolog-3 spectrometer equipped with a 450 W xenon arc lamp, double-grating excitation and emission monochromators (2.1 nm·mm^–1^ dispersion; 1200 grooves per mm), and a Hamamatsu model R928 photomultiplier tube. For Na-EuSAP and Na-GdEuSAP samples, excitation spectra were monitored at 615 nm, while emission spectra were recorded under irradiation at 273 and 395 nm. The samples analyzed were characterized at the solid state and in aqueous suspension (5 mg/mL, in the presence of 0.1 wt % of xanthan gum). Time-resolved measurements were performed using the time-correlated single-photon counting option. A 370 nm SpectraLED laser was used to excite Na-EuSAP and Na-GdEuSAP, monitoring the emission band of Eu^3+^ at 615 nm. Signals were collected using an IBH DataStation Hub photon counting module. Data analysis was performed using commercially available DAS6 software (HORIBA Jobin Yvon IBH).

The water proton longitudinal relaxation rates (*R*_1_ = 1/T_1_) were measured using a variable-field relaxometer equipped with an HTS-110 3 T metrology cryogen-*free* superconducting magnet (Mede, Italy), operating in the overall range of proton Larmor frequencies of 20–120 MHz (0.47–3.00 T). The measurements were performed using the standard inversion recovery sequence (20 experiments, two scans) with a typical 90° pulse width of 3.5 μs, and the reproducibility of the data was within ±0.5%. The temperature was controlled with a Stelar VTC-91 heater airflow equipped with a copper–constantan thermocouple (uncertainty of ±0.1 °C). Additional points in the 0.01–10 MHz frequency range were collected on a fast-field cycling Stelar SmarTracer relaxometer. Na-GdEuSAP and Na-GdSAP solids (10 mg) were dispersed in 1.5 mL of ultrapure water in the presence of xanthan gum (0.1 wt %) to improve particle dispersion. Before measurements, the suspensions were sonicated for 30 min. The pH of the suspensions was 7.0.

## 3 Results and Discussion

### 3.1 Structure and Morphology

Na-GdEuSAP and the reference samples (Na-EuSAP and Na-GdSAP) were prepared by adapting a *one-pot* hydrothermal procedure reported in the literature (Scheme S1).^22−24^ Eu^3+^ and Gd^3+^ were inserted as chloride salts during the preparation of the gel, in the presence of silicon, aluminum, and magnesium sources. The solids were then exchanged through a cationic-exchange process to replace intercalated ions (*i.e.*, Al^3+^, Mg^2+^, and H_3_O^+^) by Na^+^ ions.

The Gd^3+^ and Eu^3+^ loadings in the bifunctional GdEuSAP clay, determined by ICP–MS after mineralization of the solid in acidic solutions, were found to be *ca.* 0.03 mmol/g, prior to the treatment in the saturated solution of NaCl. No concentration change was observed after the Na^+^-exchange process, thus suggesting that both metals are in framework positions. Comparable results were also observed for GdSAP and EuSAP clays and for their parent Na^+^-exchanged samples (Na-GdSAP and Na-EuSAP, respectively) (Table S1).

**Figure 2:**
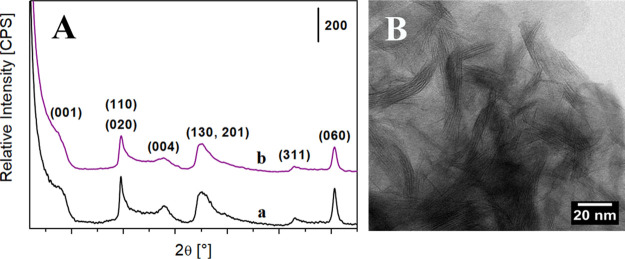
(A) X-ray patterns of Na-SAP (a) and Na-GdEuSAP (b). (B) HRTEM micrograph of Na-GdEuSAP.

The introduction of both ions did not change the inorganic structure of clay,^4,22−24^ as observed by XRPD analysis (Figures 2A and S1). Indeed, the X-ray pattern of the Na-GdEuSAP sample showed the reflections of a Na^+^-exchanged saponite.^1,27^

The morphological features of the bifunctional clay were evaluated by HRTEM (Figure 2B). The Na-GdEuSAP sample showed different distributions of the lamellae, from isolated sheets to aggregates of tactoids with different particle sizes. The introduction of both Gd^3+^ and Eu^3+^ ions in the saponite framework affected the size of lamellae, that is comparable to what was observed for Nb^5+^ and V^3+^-containing saponites prepared with the same synthetic procedure.^22−24^ Similar results in terms of morphology and particle size were also observed for the reference monofunctionalized solids (Figure S2).

The CEC values, determined by a well-known UV–Vis method (Figure S3),^28,29^ are comparable for the three functionalized clays: 44.7 ± 7.9, 45.1 ± 3.5, and 43.0 ± 5.7 mequiv/100 g for Na-GdEuSAP, Na-EuSAP, and Na-GdSAP, respectively.

DLS analyses were also performed (Figure S4) to characterize the hydrodynamic diameter of the nanoparticles dispersed in aqueous solution. The samples were dispersed in water in the presence of 0.1 wt % of xanthan gum,^16,30^ and the measures were collected at 25 °C. The dispersions were stable and homogeneous, with particle sizes characterized by the hydrodynamic diameter in the 50–60 nm range (Figure S4).

### 3.2 Photophysical Properties

The photophysical properties of the bifunctional Na-GdEuSAP clay, both in H_2_O suspension and in the form of powder, were studied by steady-state PL spectroscopy, thus obtaining insights into the coordination sphere of the structural Eu^3+^ sites. The Na-EuSAP sample has been used as a reference material (Figures S5–S7).

**Figure 3:**
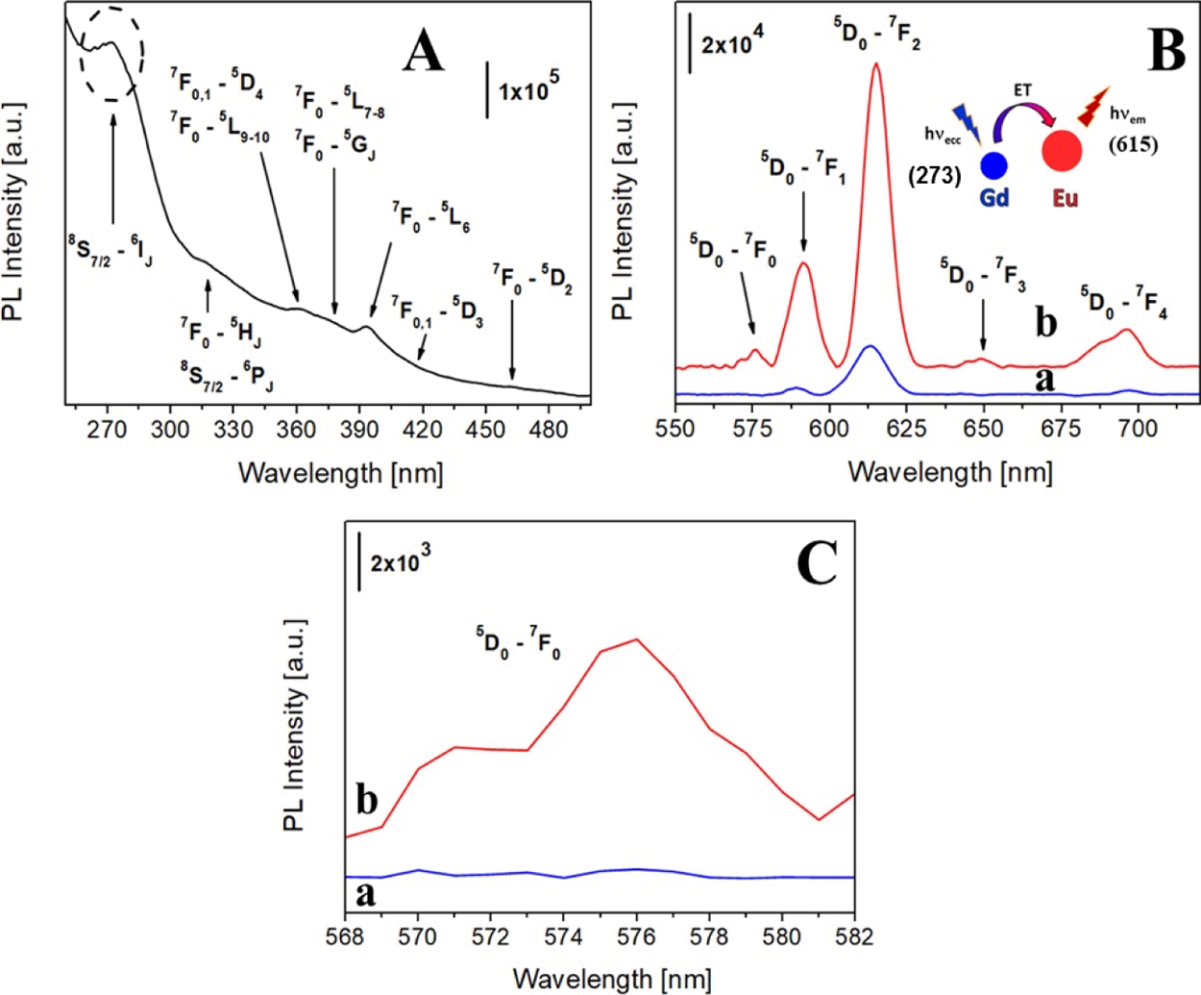
(A) Excitation spectrum in the aqueous suspension of Na-GdEuSAP. (B) PL spectra in the aqueous suspension of Na-GdEuSAP, under excitation at 395 (a) and 273 nm (b). (C) Magnification of the PL spectra shown in panel (B), in the 568–582 nm range.

The excitation spectrum of Na-GdEuSAP in aqueous suspension (Figure 3A), collected by analyzing the most intense band of Eu^3+^ at 615 nm, showed the characteristic peaks of the intra-4f^6^ electronic transitions of Eu^3+^ (^7^F_0,1_–^5^H_*J*_, ^5^D_*J*_, ^5^L_*J*_, and ^5^G_*J*_) with the main peak at 395 nm ascribed to ^7^F_0_–^5^L_6_.^31−33^ Additional signals attributed to the ^8^S_7/2_–^6^I_*J*_ (λ_max_ = 273 nm) and ^8^S_7/2_–^6^P_*J*_ electronic transitions of Gd^3+^ are also detectable.^34^ The presence of these peaks typical of Gd^3+^ is indicative of a Gd^3+^ → Eu^3+^ energy transfer process, due to a partial overlapping of the energy levels of the two metals, as indicated in the Jablonski energy diagrams.^35^ The same transitions were also observed in the spectrum of the powder sample (Figure S8).

The PL spectra of the aqueous suspension of Na-GdEuSAP, collected under excitation at both 395 nm (λ_max_ of Eu^3+^) and 273 nm (λ_max_ of Gd^3+^) (Figure 3B (a, b)), showed the typical peaks of the intra-4f^6^ electronic levels of Eu^3+^ (^5^D_0_–^7^F_*J*_, *J* = 0–4).^31−33^ The analysis of the 570–580 nm range reveals two bands at 571 and 576 nm assigned to the ^5^D_0_–^7^F_0_ transition (Figure 3C), mainly visible after irradiation at 273 nm. The splitting of this band suggests the presence of two chemically distinct environments of Eu^3+^.^36^ It is important to note that the direct excitation of Gd^3+^ at 273 nm promoted an evident increase in the intensity of all the emissions peaks of Eu^3+^. An enhancement of *ca.* 550% of the intensity of the emission band assigned to the ^5^D_0_–^7^F_2_ transition at 615 nm was observed. This result is assigned to the occurrence of the Gd^3+^ → Eu^3+^ energy transfer process.^35^

The intensity ratio of the bands at 615 (assigned to the electric dipole ^5^D_0_ → ^7^F_2_ transition) and 592 nm (the magnetic dipole ^5^D_0_ → ^7^F_1_ transition), defined by the asymmetry factor (*R*) parameter, is related to the symmetry of the coordination environment around the Eu^3+^ centers.^32,33,37−39^*R* can be 0 in the case of symmetric Eu^3+^ sites, whereas it can be higher in the case of ions with lower symmetry.^38^ The *R* factors calculated for Na-GdEuSAP, both in the aqueous suspension and in the powder form for both excitation wavelengths, assume values greater than 2, thus indicating a highly asymmetrical local environment surrounding the Eu^3+^ centers (Table S2).

The average hydration state of Eu^3+^ (*q*^Eu^) was determined by the analysis of the experimental lifetimes (τ) in H_2_O and D_2_O through time-resolved fluorescence spectroscopy (Figure S9). The decay curves of the ^5^D_0_ excited state, obtained by following the decrease in the intensity of the transition at 615 nm under excitation at 370 nm, were analyzed using a bi-exponential function. A *q*^Eu^ value of *ca.* 4, calculated by eq 1,^40^ was calculated for Na-GdEuSAP (Table S2, compatible with the presence of Eu^3+^ in the tetrahedral layers of saponite). A similar hydration state was observed for the Na-EuSAP sample (Table S2).
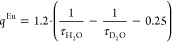
1

Photobleaching tests for Na-GdEuSAP were performed under continuous irradiation at 395 (a) and 273 nm (b) for 1 h, monitoring the intensity of the 615 nm band (Figure S10). The samples showed good photostability both in the powder form and in the water suspension.

### 3.3 Relaxometric Characterization

The ^1^H NMR relaxometric study of the Na-GdEuSAP aqueous suspension (in the presence of 0.1 wt % xanthan gum) was performed by analyzing the relaxivity values by changing the magnetic field strength and the temperature.^4,41,42^ For this purpose, Na-GdSAP was used as a reference material. The relaxivity (*r*_1p_) parameter is calculated by measuring the relaxation rate of the water protons (*R*_1_) in the presence of the paramagnetic center and dividing the obtained value by the Gd^3+^ concentration (mM).

**Figure 4:**
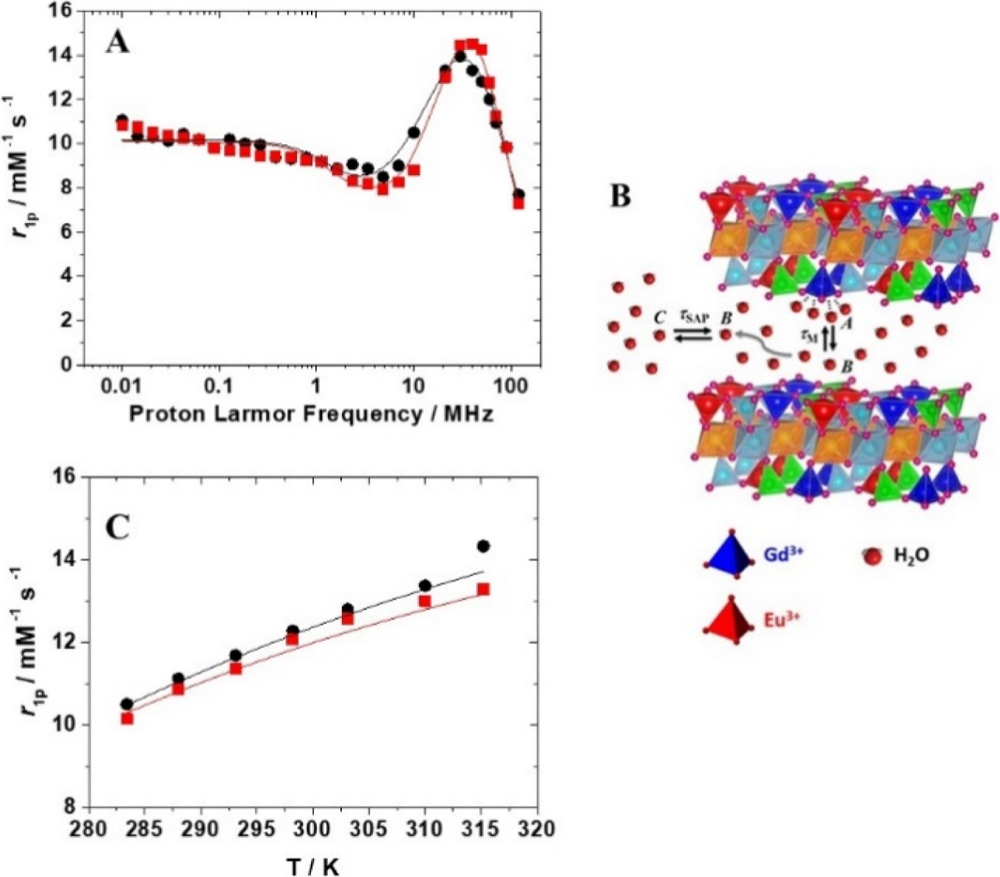
(A) 1/*T*_1_^1^H NMRD profiles of Na-GdSAP (black circles) and Na-GdEuSAP (red squares) at 37 °C, in the 0.01–120 MHz range and neutral pH. (B) Schematic representation of the water-exchange process occurring in the gallery of saponite. (C) Variable-temperature dependence of *r*_1p_ for Na-GdSAP (black circles) and Na-GdEuSAP (red squares), at 20 MHz.

^1^H 1/*T*_1_ nuclear magnetic relaxation dispersion (NMRD) profiles of both Na-GdSAP and Na-GdEuSAP, collected in the 0.01–120 MHz range at 37 °C and neutral pH (Figure 4A (a, b)), show the typical shape of systems with slow rotation,^43^ with a hump centered at *ca.* 40 MHz. The relaxivity calculated at the clinical field (1.5 T) is 12.7 mM^–1^ s^–1^, about three times higher than that of the clinically used Gd^3+^ chelates^4^ and comparable to that of some Gd^3+^-doped microporous zeolites.^44^

A quantitative analysis of the observed relaxivities is rather cumbersome, since *r*_1p_ values are affected by a rather large number of parameters. In fact, the inner-sphere water molecules should be involved in chemical exchange with the surrounding water molecules in the interlayer space. Subsequently, they must diffuse through the interlayer space to reach the bulk water. For microporous zeolites, which are well-characterized systems expected to behave similarly to saponite clays, the observed relaxivities are analyzed using a two-step exchange model, where water molecules bound to Gd^3+^ in the pores are in exchange with the bulk H_2_O. A characteristic feature of microporous zeolites is that the observed relaxivity decreases upon increasing Gd^3+^ loading. This effect is due to a reduction in the hydration state of the Gd^3+^ ions inside the pores.^44^ However, the Gd^3+^ ions in Na-GdSAP and Na-GdEuSAP are expected to be exposed to a relatively large water content in the interlayer space, rather than in a confined environment. Thus, it is reasonable to assume that the residence time of inner-sphere water (τ_m_) is much shorter than the mean residence time of water molecules in the interlamellar space (τ_SAP_). The slow diffusion of water molecules through the interlayer space is therefore expected to limit the water ^1^H relaxivity. The temperature dependence of *r*_1p_ measured at 20 MHz (Figure 4C) supports this hypothesis, since an increase in *r*_1p_ at higher temperatures is observed as a result of a shorter τ_SAP_.

In a first attempt, the ^1^H NMRD profiles recorded for Na-GdSAP and Na-GdEuSAP were analyzed using Solomon–Bloembergen–Morgan theory of paramagnetic relaxation.^45^ Since the Gd^3+^ ions are immobilized in the clay framework, we assumed that the outer-sphere effect, arising from bulk H_2_O diffusing in the vicinity of the paramagnetic ions, can be neglected. However, the data fitted using this model provided unrealistically low activation energies for τ_SAP_ (∼3 kJ·mol^–1^). Indeed, activation energies in the range 10–20 kJ·mol^–1^ were reported for synthetic saponites having different hydration states, chemical natures of cations, and charges of the tetrahedral layer.^46^ This supports that the activation energy should not differ considerably from that measured for the diffusion of H_2_O in pure water (17.6 kJ·mol^–1^)^47^ and indicates that the local mobility of water molecules bonded to the Gd^3+^ ion is limiting the relaxivity to a certain extent. Thus, we attempted quantitative fitting of the data using the Lipari–Szabo model,^48^ which considers both local (τ_RL_) and global (τ_RG_) rotational correlation times. The distance between the protons of the coordinated water molecules and the Gd^3+^ ion (*r*_GdH_) was assumed to be 3.1 Å.^49^ The number of inner-sphere water molecules was set to four, considering a hydration state of Gd^3+^ comparable to that of Eu^3+^, estimated by luminescence data. The experimental data could be fitted very well with this simple model, providing essentially the same parameters for Na-GdSAP and Na-GdEuSAP (Table 1).

**Table 1:** Parameters Derived from the Analysis of ^1^H NMRD Profiles for Na-GdSAP and Na-GdEuSAP

parameter	Na-GdSAP	Na-GdEuSAP
τ_SAP_^298^/μs	2.4 ± 0.2	2.3 ± 0.2
Δ*H*^⧧^/kJ·mol^–1^	17.6^a^	17.6^a^
τ_RL_^298^/ps	34 ± 2	32 ± 2
τ_RG_^298^/ns	2.2 ± 0.1	2.9 ± 0.2
*S*^2^	0.088 ± 0.003	0.088 ± 0.004
*E*_r_/kJ·mol^–1^	22^a^	22^a^
τ_v_/ps	49 ± 2	35 ± 2
Δ^2^/10^19^ s^–2^	1.7 ± 0.1	2.4 ± 0.1
*r*_GdH_/Å	3.1^a^	3.1^a^
*q*^298^	4^a^	4^a^

a Parameters set as constant during the fitting procedures.

The analysis of the data indicated a τ_sap_ of water molecules inside the clay of about 2 μs. Longer average residence times of H_2_O entrapped in the interlayer space of synthetic fluorohectorite were determined in the solid state.^50^ However, the residence time may be shorter in the water suspension. Moreover, the residence time is also expected to be affected by the size of the particles. We also point out that Gd^3+^ ions at longer distances from the bulk water may be silent or provide a smaller contribution to the observed relaxivity, which may be dominated by the contribution of paramagnetic centers exposed to the bulk water. The analysis of the data afforded a long τ_RG_ value of 2–3 ns and a short correlation time of τ_RL_ = ∼35 ps. The low value of *S*^2^, which is typically between 0 and 1, suggests that relaxivity is limited by the high fast local mobility. The parameters that determine the relaxation of the electron spin, the mean square zero-field splitting energy (Δ^2^), and the correlation time for the zero-field splitting interaction (τ_v_) fall within the range usually determined for Gd^3+^ compounds,^51^ which provides confidence in the results of the analysis.

**Figure 5:**
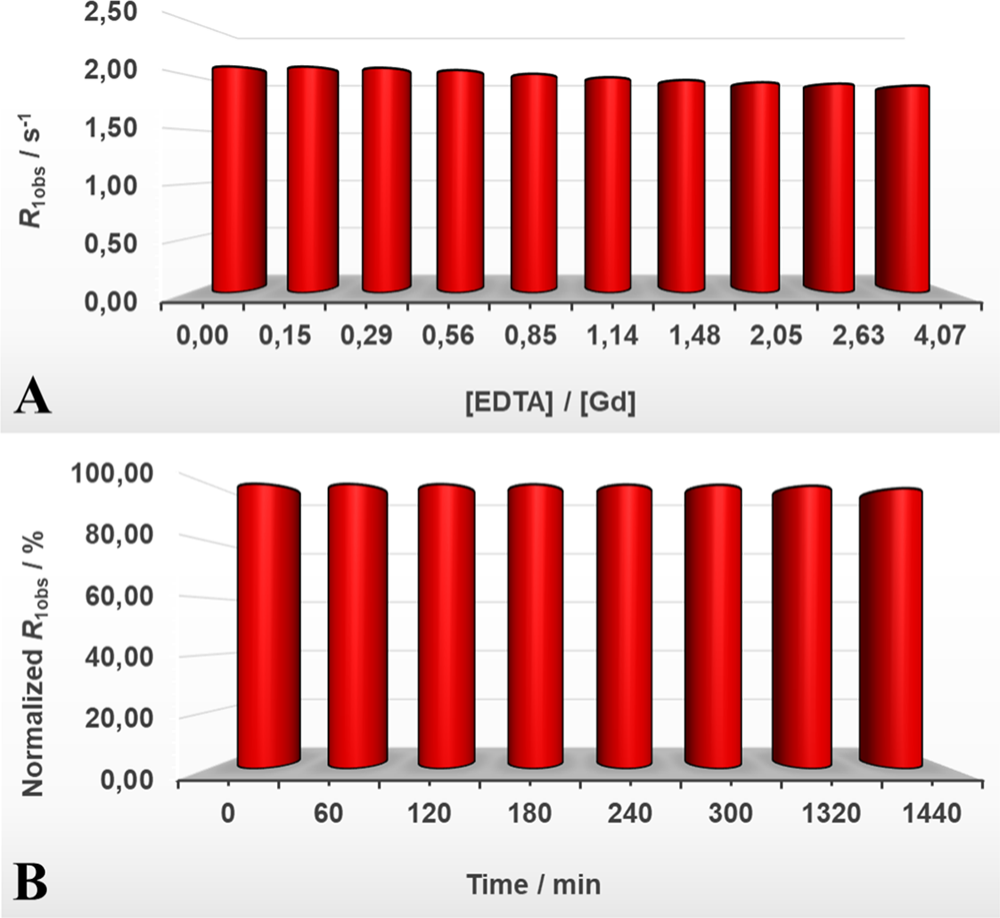
*R*_1_ value (20 MHz and 25 °C) for Na-GdEuSAP treated with an increasing amount of EDTA (A) over time (with [EDTA]/[Gd] = 4) (B).

Finally, the stability of aqueous suspensions and the chemical integrity of the bifunctional solid have been evaluated under different conditions, monitoring the relaxation values over time. The same studies were also conducted on Na-GdSAP (Figures S11 and S12). The *r*_1p_ value of the aqueous suspension of Na-GdEuSAP at 20 MHz and 25 °C remains constant for up to 24 h, indicating the absence of particle sedimentation and thus suggesting a good stability of the suspension (Figure S11). To gain insights into the chemical integrity of the bifunctional clay, the dispersion was then treated with an increasing amount of the ethylenediaminetetraacetic acid (EDTA) (Figure 5). The *R*_1_ value (25 °C and 20 MHz) did not change under different concentrations of EDTA, up to 1:1 ratio of EDTA/Gd^3+^ (Figure 5A), indicating the absence of Gd^3+^ release in solution, under these conditions. Under extreme conditions, in large excess of EDTA (EDTA/Gd = 4:1), we observed a slight decrease in the relaxation rate (*ca.* 9%), associated to a release of Gd^3+^ of 23% (Figure 5A). No further metal leaching was detected over time (Figure 5B).^52^ The same performance was also observed for the reference material (Figure S12A,B). These data are a proof of the appreciable chemical stability of the bifunctional clay.

## 4 Conclusions

In conclusion, the bifunctional saponite sample studied here exhibited interesting optical and magnetic properties associated to the co-presence of Eu^3+^ and Gd^3+^ ions in structural positions. The high relaxivity at clinical fields with respect to the commercially available chelates is due to the high hydration state of Gd^3+^ and to a reduced mobility of the functionalized nanoparticles in aqueous media. However, the local mobility of the inner-sphere water molecules and their slow diffusion along the interlayer space limit relaxivity to a certain extent. Furthermore, the optical properties of the solid can be modulated through a direct excitation of Eu^3+^ or by promoting energy transfer from Gd^3+^ to Eu^3+^ located in the inorganic framework.

Finally, the bifunctional clay showed good stability both in pure water and after addition of an equimolar amount of a strong chelating agent. This confirms the high robustness of these solids and the structural confinement of the lanthanide ions into the lamellae. These properties thereby make it a desirable platform for diagnostic applications. Additional studies on biological matrices are planned in the future.

## Supporting Information Available

Characterization of the clays by powder X-ray diffraction, TEM, UV–Vis, and DLS, additional photophysical studies, luminescence decay profiles, and equations used to fit the relaxivity data (PDF)
