# Supplementary material for: Bifunctional Paramagnetic and Luminescent Clays Obtained by Incorporation of Gd3+ and Eu3+ Ions in the Saponite Framework
Source: Inorg Chem. 2021 Jul 9;60(14):10749–56. doi: 10.1021/acs.inorgchem.1c01455 (PMC8389799; doi:10.1021/acs.inorgchem.1c01455)
Supplement: Supplementary file 1 — ic1c01455_si_001.pdf [file ic1c01455_si_001.pdf]

ELECTRONIC SUPPLEMENTARY INFORMATION

Bifunctional paramagnetic and luminescent clays obtained by incorporation of Gd^{3+} and Eu^{3+} ions in the saponite framework

Stefano Marchesi,[a] Chiara Bisio,[a,b] Daniela Lalli,[a] Leonardo Marchese,[a] Carlos Platas-Iglesias,[c] and Fabio Carniato[a]**

[a] Dipartimento di Scienze e Innovazione Tecnologica, Università degli Studi del Piemonte Orientale "Amedeo Avogadro", Viale Teresa Michel 11, 15121-Alessandria (Italy)

[b] CNR-SCITEC Istituto di Scienze e Tecnologie Chimiche "G. Natta", Via C. Golgi 19, 20133-Milano (Italy)

[c] Centro de Investigacións Científicas Avanzadas (CICA) and Departamento de Química, Facultade de Ciencias, Universidade da Coruña, 15071 A Coruña, Galicia (Spain)

*E-mail: fabio.carniato@uniupo.it; chiara.bisio@uniupo.it

*Fax: +39 0131360250; Tel: +39 0131360217, +39 0131360216

1) FIGURES

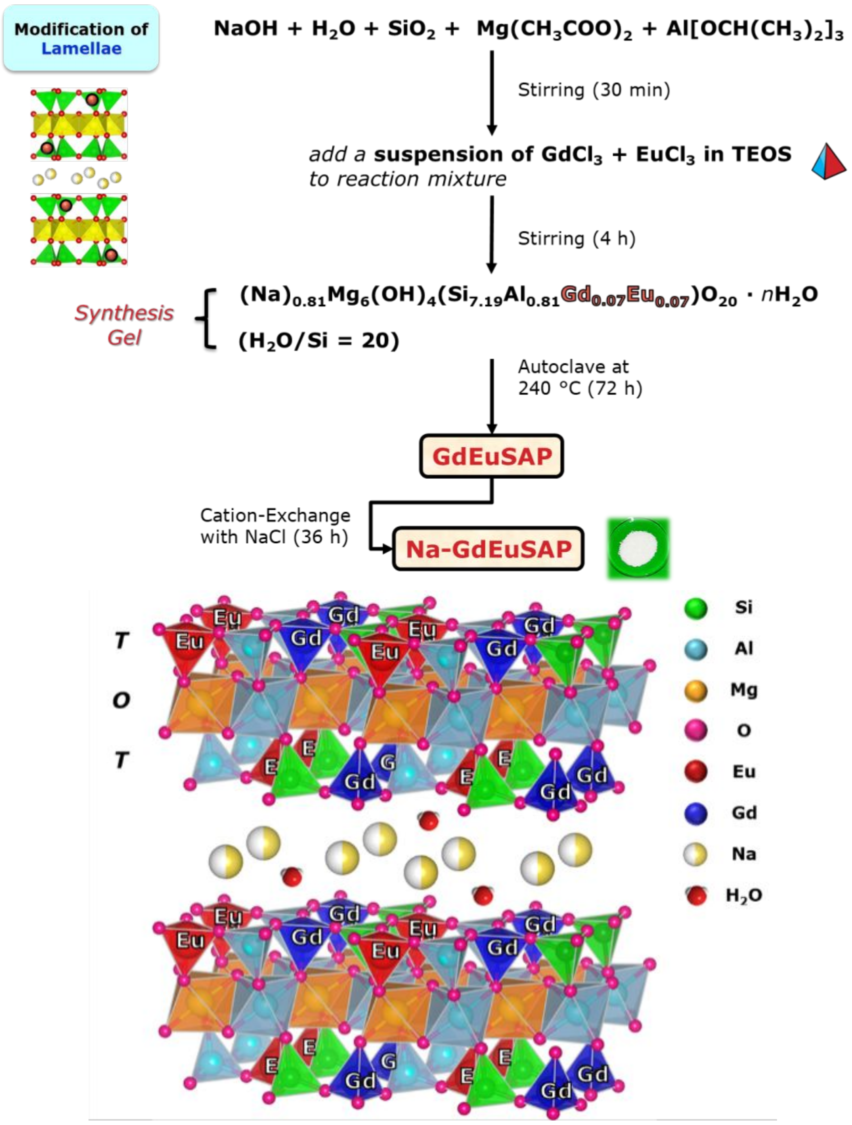

Scheme S1. Schematic representation of the preparation procedure of the bifunctional Na-GdEuSAP clay and related structure.

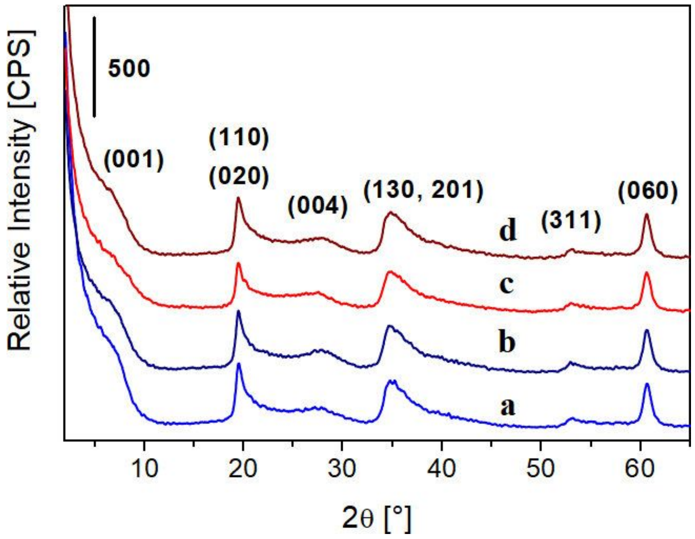

Figure S1. X-ray profiles of GdSAP (a), EuSAP (b), Na-GdSAP (c) and Na-EuSAP (d).

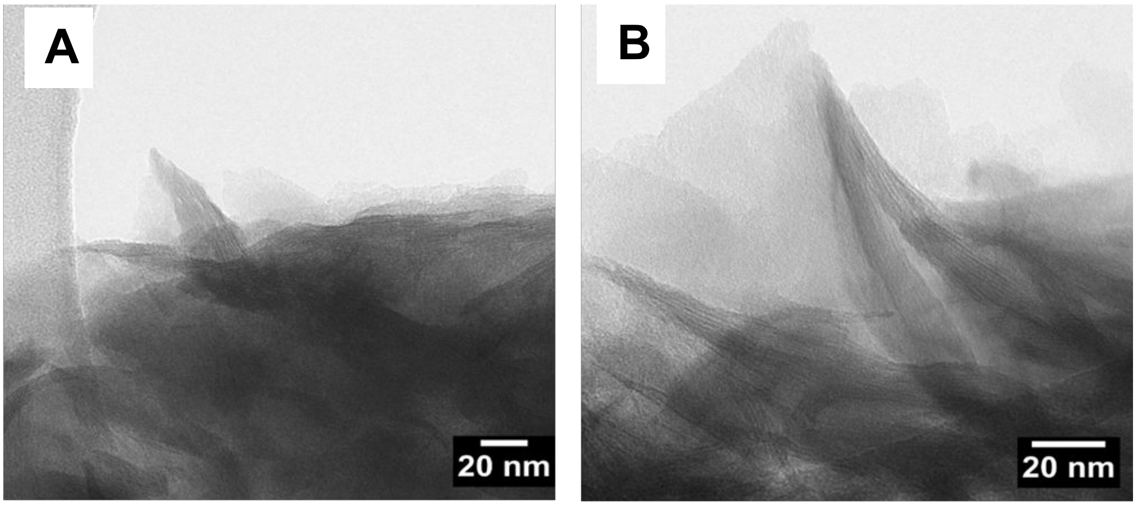

Figure S2. HRTEM micrographs of Na-GdSAP (A) and Na-EuSAP (B).

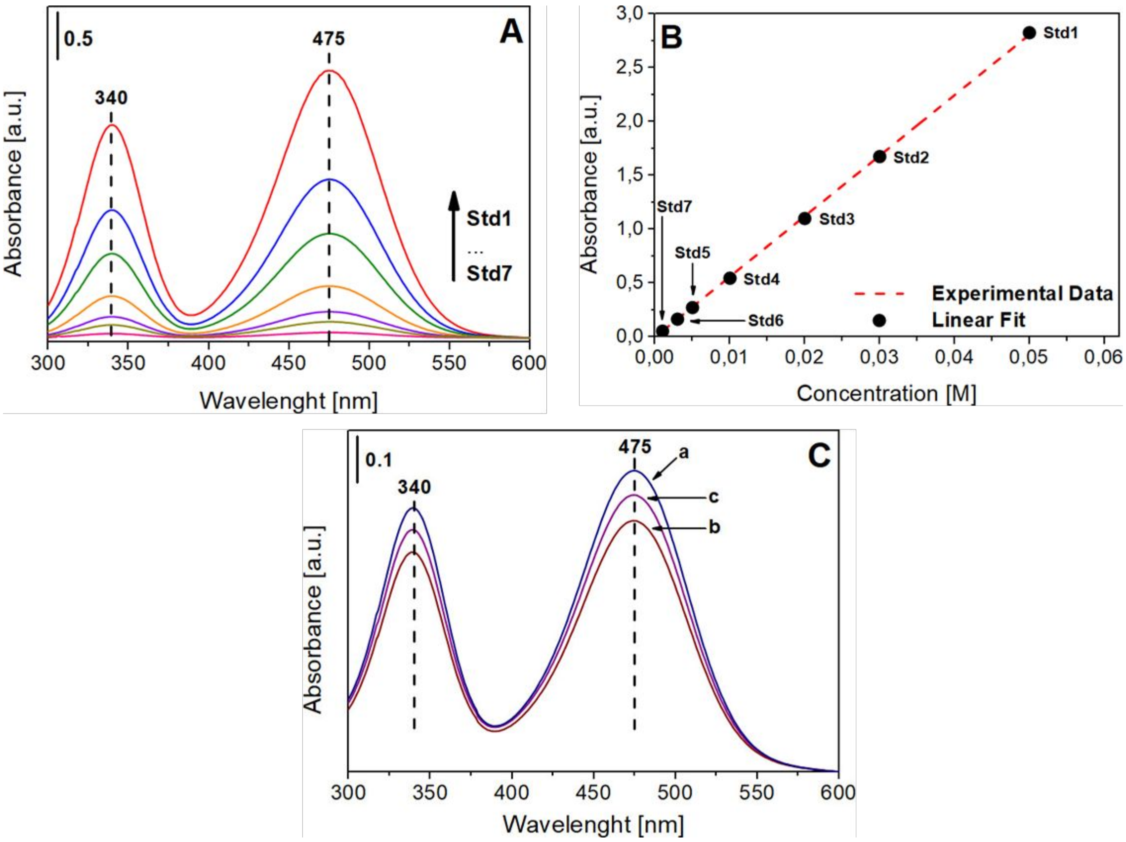

Figure S3. A) UV-Vis spectra of standard solutions of $[Co(NH_3)_6]^{3+}$, from 0.05 mM to 0.001 mM (Std1 to Std7); B) Calibration curve obtained by UV-Vis spectra (λ_{abs} = 475 nm) of $[Co(NH_3)_6]^{3+}$ in H_2O at room temperature; C) UV-Vis spectra of the supernatant of Co-GdSAP (a), Co-EuSAP (b) and Co-GdEuSAP (c) test samples.

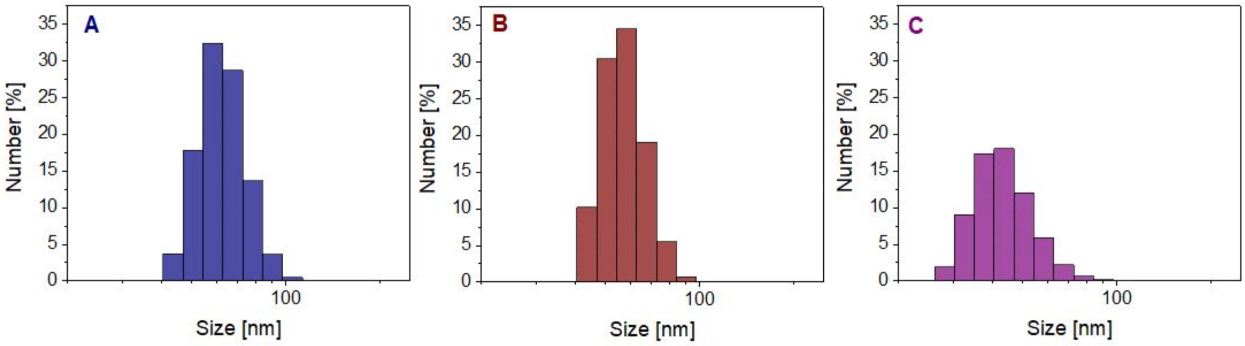

Figure S4. Hydrodynamic diameters distribution in aqueous solution of Na-GdSAP (A), Na-EuSAP (B) and Na-GdEuSAP (C), at 25 °C.

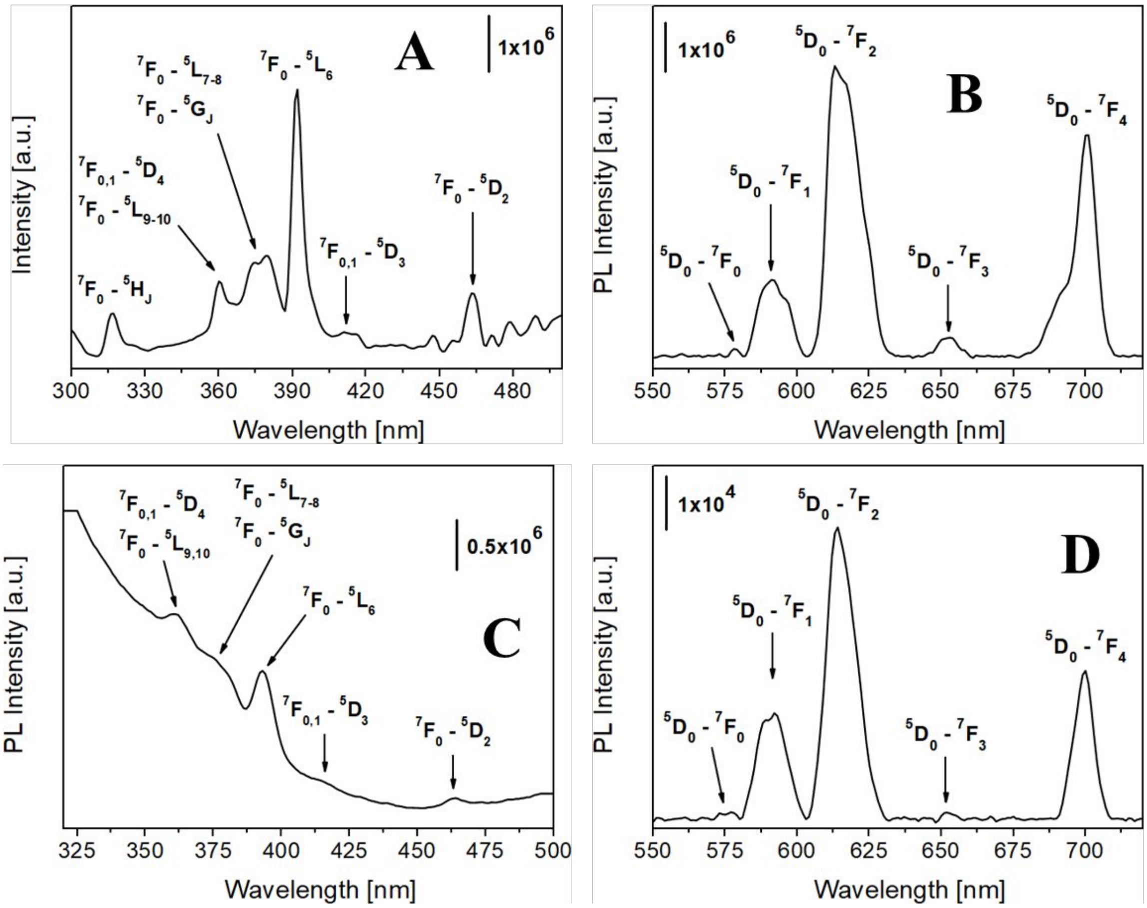

Figure S5. Excitation spectra at solid state (A) and in aqueous suspension (C) of Na-EuSAP, collected at 615 nm. Emission spectra at solid state (B) and in aqueous suspension (D) of Na-EuSAP, under excitation at 395 nm.

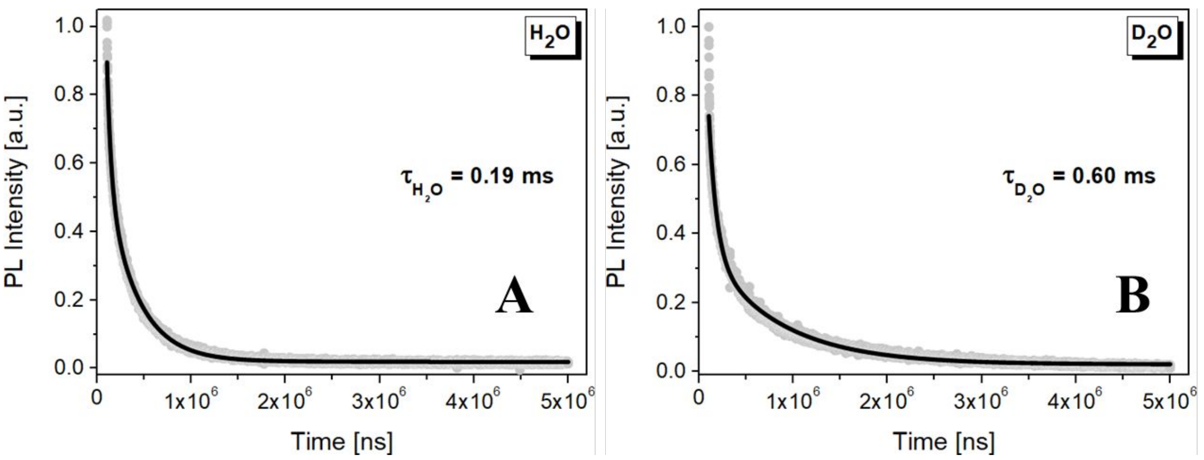

Figure S6. PL $^5D_0$-$^7F_2$ (615 nm) intensity decay profiles over time of Na-EuSAP dispersed in H_2O (A) and D_2O (B), under irradiation at 370 nm with a SpectraLED laser source. The fitting was performed with a bi-exponential function (black lines). The χ^2 and *RSS* (residual sum of squares) values are reported in the table below:

**Sample**	χ^2	***RSS***
Na-EuSAP, H_2O	3.7954E-5	0.37096
Na-EuSAP, D_2O	8.5264E-5	0.83337

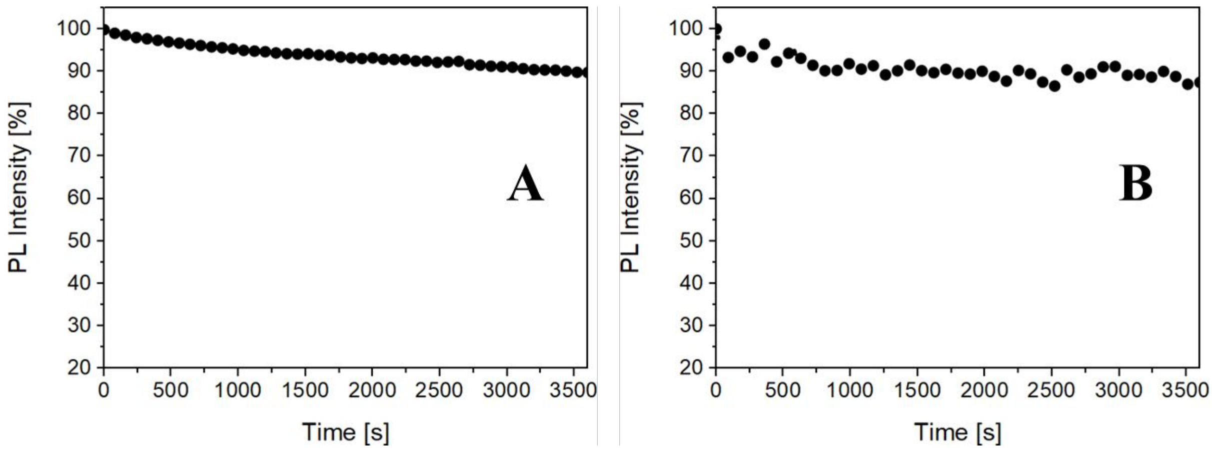

Figure S7. Photobleaching tests of Na-EuSAP at solid state (A) and in aqueous suspension (B), under irradiation at 395 nm. The intensity of $^5D_0$-$^7F_2$ transition (615 nm) was monitored for 1 h.

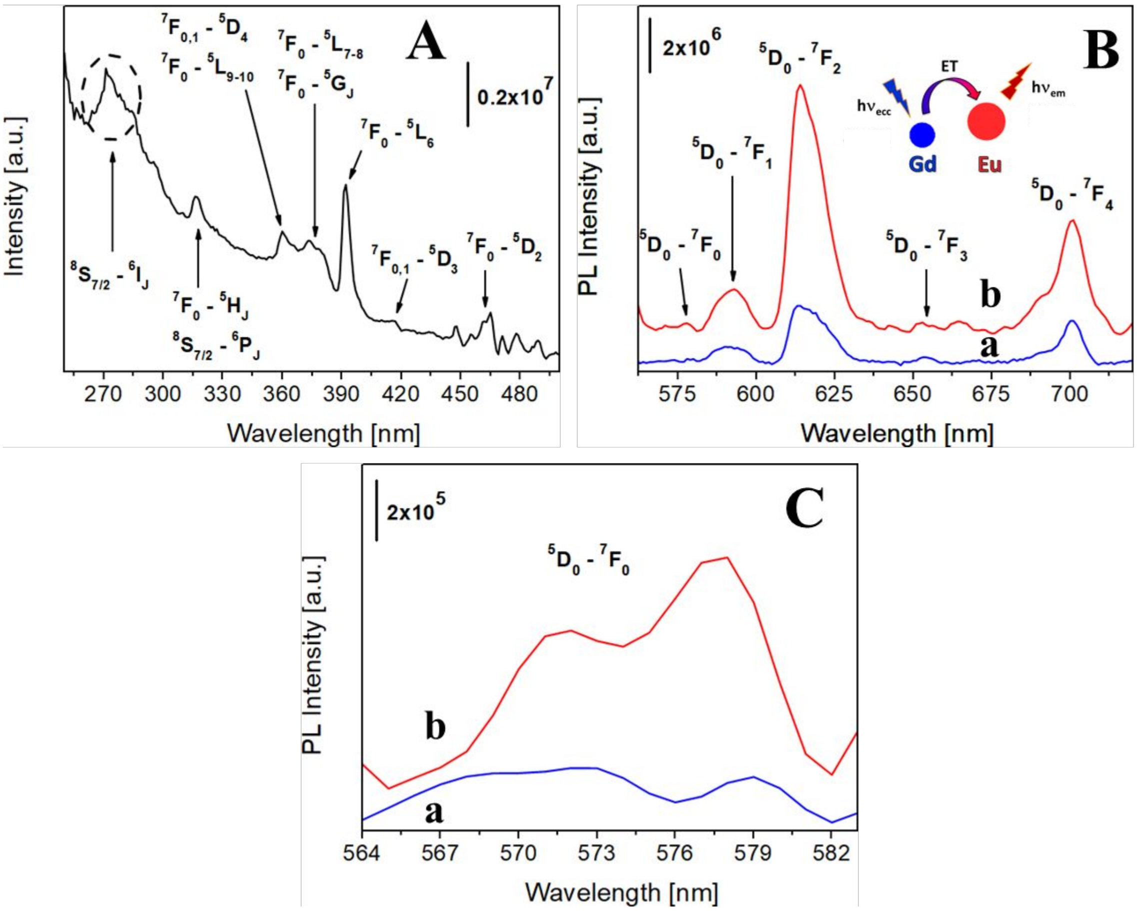

Figure S8. A) Excitation spectrum at solid state of Na-GdEuSAP, collected at 615 nm. B) Emission spectra at solid state of Na-GdEuSAP, under excitation at 395 (a) and 273 nm (b). A magnification of spectra in the 564-583 nm range is shown in C.

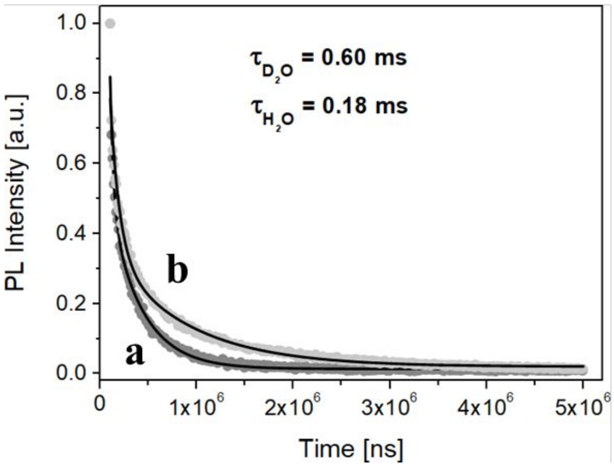

Figure S9. Normalized PL $^5D_0$-$^7F_2$ (615 nm) intensity decay profiles over time of Na-GdEuSAP dispersed in H_2O (a) and D_2O (b), under irradiation at 370 nm with a SpectraLED laser source. The fitting was performed with a bi-exponential function (black lines). The χ^2 and *RSS* (residual sum of squares) values of the lifetime fitting in H_2O and D_2O are reported in the table below:

**Sample**	χ^2	***RSS***
Na-GdEuSAP, H_2O	4.3452E-5	0.42492
Na-GdEuSAP, D_2O	9.0927E-5	0.88853

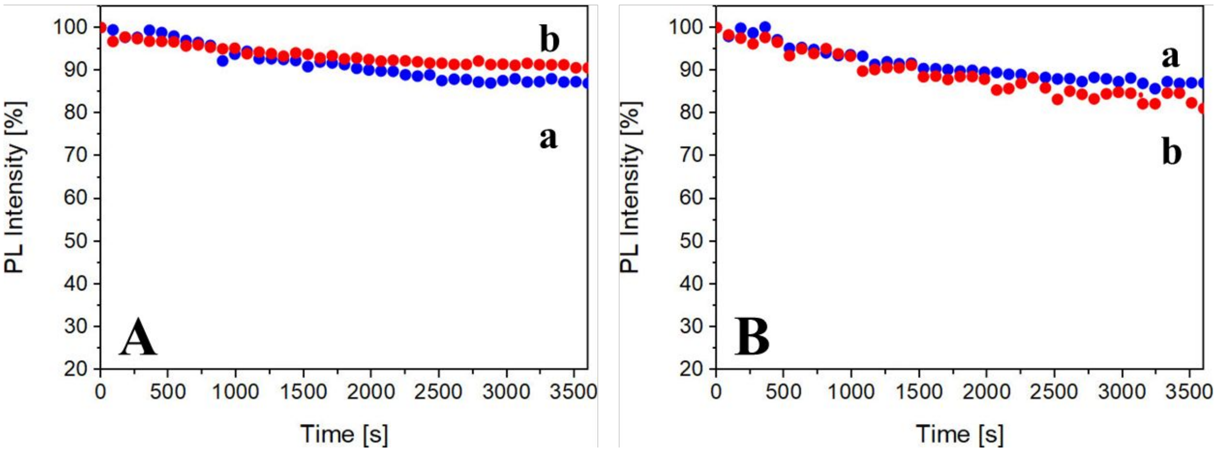

Figure S10. Photobleaching tests of Na-GdEuSAP at solid state (A) and in aqueous suspension (B), under irradiation at 395 (a) and 273 (b) nm. The intensity of $^5D_0$-$^7F_2$ transition (615 nm) was monitored for 1 h.

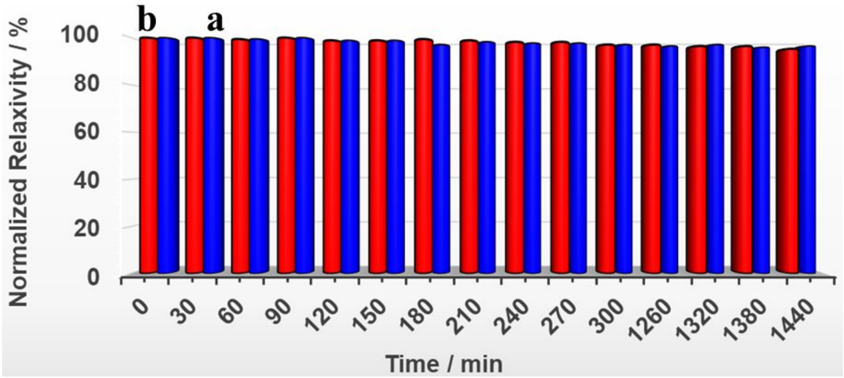

Figure S11. Histograms of normalized r_1 % at 20 MHz and 25 °C of Na-GdSAP (blue, a) and Na-GdEuSAP (red, b) in aqueous suspension over time.

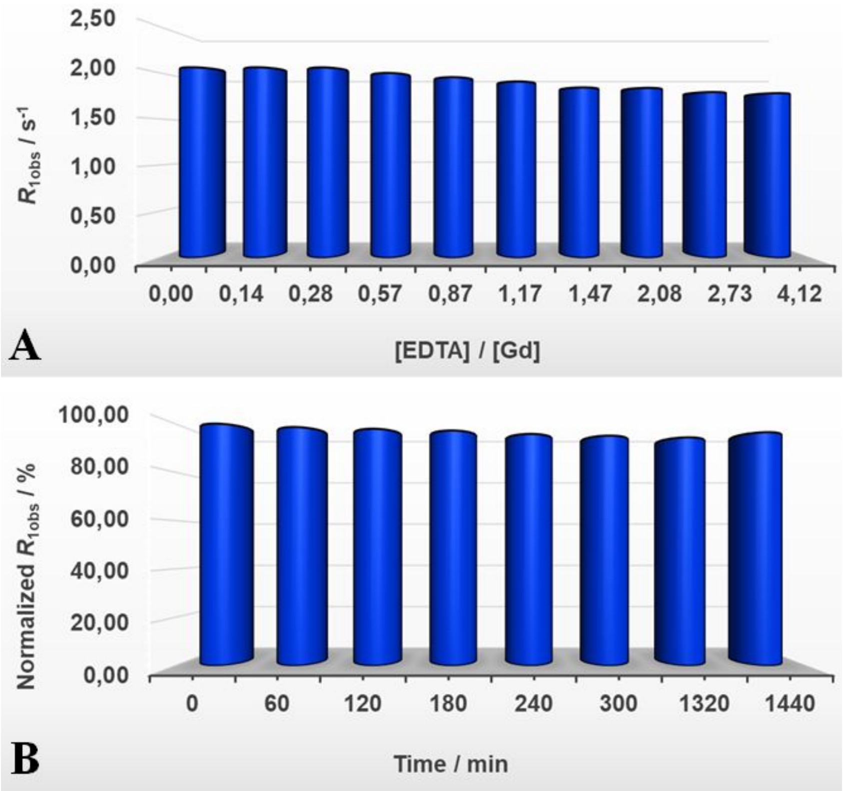

Figure S12. Relaxation rate values at 20 MHz and 25 °C for Na-GdSAP treated with increasing amounts of EDTA (A) and over time (with [EDTA]/[Gd] = 4.12) (B).

2) TABLES

Table S1. Eu^{3+} and Gd^{3+} loadings in the samples before (GdSAP, EuSAP and GdEuSAP) and after Na^{+}-exchange process (Na-GdSAP, Na-EuSAP and Na-GdEuSAP), obtained by ICP-MS elemental analyses.

Sample	Eu^{3+} [mmol/g]	Gd^{3+} [mmol/g]
GdSAP	/	$2.55\cdot10^{-2}$
Na-GdSAP	/	$2.52\cdot10^{-2}$
EuSAP	$2.71\cdot10^{-2}$	/
Na-EuSAP	$2.69\cdot10^{-2}$	/
GdEuSAP	$2.61\cdot10^{-2}$	$2.63\cdot10^{-2}$
Na-GdEuSAP	$2.60\cdot10^{-2}$	$2.61\cdot10^{-2}$

Table S2. Photophysical parameters of Na-GdEuSAP and Na-EuSAP samples, in the solid state and in aqueous suspensions.

	Na-EuSAP		Na-GdEuSAP			
Asymmetry Factor (R)	Solid	H_2O	Solid (λ_{exc}=273 nm)	Solid (λ_{exc}= 395 nm)	H_2O (λ_{exc}=273 nm)	H_2O (λ_{exc}=395 nm)
	3.10	2.76	3.15	3.10	2.52	2.57
τ_{H2O} [ms]	0.19		0.18			
τ_{D2O} [ms]	0.60		0.60			
q^{Eu} [a]	4.02		4.37			

[a] calculated from Eq[1]: $q^{Eu} = 1.2 \cdot \left({}^{1}/_{\tau_{H_2O}} - {}^{1}/_{\tau_{D_2O}} - 0.25 \right)$

3) ^{1}H-NMRD

The relaxivity r_{1p} can be divided into inner- ($r_{1p,is}$) and outer-sphere ($r_{1p,os}$) contributions:

$$r_{1p} = r_{1p,is} + r_{1p,os} \quad (1)$$

The outer-sphere contribution is described by the Freed's model reported in the following equation:[2,3]

$$r_{1p,OS} = \frac{32 N_A \pi}{405} \left(\frac{\mu_0}{4\pi} \right)^2 \frac{\hbar^2 \gamma_S{}^2 \gamma_I{}^2}{a_{GdH} D_{GdH}} S(S+1) [3 J_{OS}(\omega_I; T_{1e}) + 7 J_{OS}(\omega_I; T_{2e})] \quad (2)$$

$$J_{OS}(\omega_I; T_{je}) = Re \left[\frac{1 + \frac{1}{4}\left(i\omega\tau_{GdH} + \frac{\tau_{GdH}}{T_{je}} \right)^{1/2}}{1 + \left(i\omega\tau_{GdH} + \frac{\tau_{GdH}}{T_{je}} \right)^{1/2} + \frac{4}{9}\left(i\omega\tau_{GdH} + \frac{\tau_{GdH}}{T_{je}} \right) + \frac{1}{9}\left(i\omega\tau_{GdH} + \frac{\tau_{GdH}}{T_{je}} \right)^{3/2}} \right] \quad (3)$$

$$\tau_{GdH} = \frac{a_{GdH}{}^2}{D_{GdH}} \quad (4)$$

N_A is the Avogadro constant, a_{GdH} is the distance of closest approach of an outer-sphere water molecule to the Gd center, ω_I is the nuclear Larmor frequency, T_{1e} and T_{2e} are the longitudinal and transverse relaxation times of the electron spin, S is the electron spin, γ_I and γ_S are the nuclear and electron gyromagnetic ratios, and D_{GdH} is the relative translational diffusion coefficient, calculated as the sum of the self-diffusion coefficients of the Gd chelate and water molecules.

The longitudinal and transverse electronic relaxation rates are approximated by Eqs (5)-(6), where τ_V is the electronic correlation time for the modulation of the zero-field splitting interaction, Δ^2 is the mean square zero-field-splitting energy and ω_S is the electron Larmor frequency.[4]

$$\frac{1}{T_{1e}} = \frac{1}{25}\Delta^2\tau_v\{4S(S+1)-3\}\left(\frac{1}{1+\omega_s^2\tau_v^2}+\frac{4}{1+4\omega_s^2\tau_v^2}\right) \quad (5)$$

$$\frac{1}{T_{2e}} = \frac{1}{50}\Delta^2\tau_v\{4S(S+1)-3\}\left(3+\frac{5}{1+\omega_s^2\tau_v^2}+\frac{2}{1+4\omega_s^2\tau_v^2}\right) \quad (6)$$

The inner-sphere contribution to relaxivity, $r_{1p,is}$, is directly proportional to the number of water molecules coordinated to the metal ion (*q)* as reported in the following equation:

$$r_{1p,is} = \frac{1}{1000} \times \frac{q}{55.55} \times \frac{1}{T_{1m}^H + \tau_m} \quad (7)$$

τ_m is the mean residence lifetime of a water molecule in the inner coordination sphere of the metal ion and $1/T_{1m}^H$ is the longitudinal relaxation rate of inner sphere protons, which for Gd(III) may arise from dipole-dipole (DD) mechanism according to:[5-6]

$$\left(\frac{1}{T_{1m}^H}\right)^{DD} = \frac{2}{15}\left(\frac{\mu_0}{4\pi}\right)^2\frac{\gamma_I^2 g^2\mu_B^2}{r_{GdH}^6}S(S+1)\left(\frac{3\tau_{d1}}{1+\omega_I^2\tau_{d1}^2}+\frac{7\tau_{d2}}{1+4\omega_s^2\tau_{d2}^2}\right) \quad (8)$$

$$\frac{1}{\tau_{di}} = \frac{1}{\tau_R}+\frac{1}{\tau_m}+\frac{1}{T_{ie}}, \text{ with } i = 1,2 \quad (9)$$

g is the electron g factor, μ_B is the Bohr magneton, ω_I is the nuclear Larmor frequency, r_{GdH} is the distance between the electron and nuclear spins and τ_R is the rotational correlation time.

For slowly tumbling systems, the contribution of both local and global motions can be described by the model-free Lipari-Szabo approach. In this case, the Eq. (8) can be rewritten as:[7-8]

$$\left(\frac{1}{T_{1m}^{H}}\right)^{DD} = \frac{2}{15}\left(\frac{\mu_0}{4\pi}\right)^2 \frac{\gamma_I^2 g^2 \mu_B^2}{r_{GdH}^6} S(S+1)\left[\frac{3S^2\tau_{d1g}}{1+\omega_I^2\tau_{d1g}^2} + \frac{3(1-S^2)\tau_{d1}}{1+\omega_I^2\tau_{d1}^2} + \frac{7S^2\tau_{d2g}}{1+\omega_I^2\tau_{d2g}^2} + \frac{7(1-S^2)\tau_{d2}}{1+\omega_I^2\tau_{d2}^2}\right] \quad (10)$$

$$\frac{1}{\tau_{dig}} = \frac{1}{\tau_m} + \frac{1}{\tau_{RG}} + \frac{1}{T_{ie}} \qquad i = 1,2 \quad (11)$$

$$\frac{1}{\tau_{di}} = \frac{1}{\tau_m} + \frac{1}{\tau} + \frac{1}{T_{ie}} \qquad i = 1,2 \quad (12)$$

$$\frac{1}{\tau} = \frac{1}{\tau_{RG}} + \frac{1}{\tau_{RL}} \quad (13)$$

Where τ_{RG} represents the correlation time for the motion of the whole molecule, τ_{RL} is the correlation time for the fast local motion and S^2 is the generalized order parameter.

4) REFERENCES

(1) Beeby, A.; Clarkson, I. M.; Dickins, R. S.; Faulkner, S.; Parker, D.; Royle, L.; de Sousa, A. S.; Williams, J. A. G.; Woods, M. Non-radiative deactivation of the excited states of europium, terbium and ytterbium complexes by proximate energy-matched OH, NH and CH oscillators: an improved luminescence method for establishing solution hydration states. *J. Chem. Soc., Perkin Trans.* **1999**, *2*, 493-504.

(2) Freed, J. H. Dynamic effects of pair correlation functions on spin relaxation by translational diffusion in liquids. II. Finite jumps and independent T1 processes. *J. Chem. Phys.* **1978**, *68*, 4034-4037.

(3) Koenig, S. H.; Brown III, R. D. Field-cycling relaxometry of protein solutions and tissue: Implications for MRI. *Prog. Nucl. Magn Reson. Spectrosc.* **1990**, *22*, 487-567.

(4) McLachlan, A. D. Line widths of electron resonance spectra in solution. *Proc. R. Soc. London.* **1964**, *280*, 271-288.

(5) Solomon, I.; Bloembergen, N. Nuclear Magnetic Interactions in the HF Molecule. *J. Chem. Phys.* **1956**, *25*, 261-266.

(6) Bloembergen, N.; Morgan, L. O. Proton Relaxation Times in Paramagnetic Solutions. Effects of Electron Spin Relaxation. *J. Chem. Phys*. **1961**, *34*, 842-850.

(7) Lipari, G.; Szabo, A. Model-free approach to the interpretation of nuclear magnetic resonance relaxation in macromolecules. 1. Theory and range of validity *J. Am. Chem. Soc.* **1982**, *104*, 4546-4559.

(8) Lipari, G.; Szabo, A. Model-free approach to the interpretation of nuclear magnetic resonance relaxation in macromolecules. 2. Analysis of experimental results. *J. Am. Chem. Soc*. **1982**, *104*, 4559-4570.
